# Pidotimod: the past and the present

**DOI:** 10.1186/1824-7288-39-75

**Published:** 2013-12-06

**Authors:** Gian Vincenzo Zuccotti, Chiara Mameli

**Affiliations:** 1 Department of Pediatrics, Luigi Sacco Hospital, University of Milan, Via GB Grassi 74, 20157, Milan, Italy

**Keywords:** Pidotimod, Recurrent respiratory infections, Immunostimulants

## Abstract

At the end of 1990s, acute respiratory tract infections (ARTIs) were called the 'forgotten pandemic’, with a clear dichotomy between developing and industrialised countries in mortality and morbidity, the main outcomes associated with ARTIs. This definition still applies 20 years later, when the introduction of new and safe antibiotics and vaccines has certainly contributed to controlling the most life-threatening ARTIs, but has not had a major impact on viral ARTIs in paediatric age. One functional approach to preventing and treating ARTIs is non-specifically increasing the immune response or enhancing the children’s innate defence mechanisms. Different kinds of biologically active substances – called immunostimulants – of natural and synthetic origins and with different mechanisms of action have been introduced in some countries for the prevention of ARTIs in children. Recently, research focused on one of these compounds, Pidotimod, has attempted to better clarify and define its mechanisms of action both *in vitro* and *in vivo*. In this paper, we critically examine the most recent findings on Pidotimod. Certainly the improvement of research methodology in the last 20 years and the acquired knowledge in various fields of clinical immunology should be the starting point for research on Pidotimod. Preclinical research will be essential to better understand the mechanisms of action of this compound. However, *in vivo* studies, especially randomised control trials, will be necessary to establish the real efficacy of Pidotimod in the prevention of ARTIs in paediatric age.

## Background

At the end of 1990s, acute respiratory tract infections (ARTIs) were called the 'forgotten pandemic’, with a clear dichotomy between developing and industrialised countries in mortality and morbidity, the main outcomes associated with ARTIs [1]. This definition still applies 20 years later, when the introduction of new and safe antibiotics and vaccines has certainly contributed to controlling the most life-threatening ARTIs, but have not had a major impact on viral ARTIs. Viruses are the main agents responsible for ARTIs during the paediatric age and the high number of circulating virus and the different viral sub-types result in a higher probability of experiencing frequent ARTIs during childhood [2]. These epidemiological features besides the well-known immaturity of the immune system during the first years of life and the exposure to risk factors (air pollution, parental tobacco smoke, daycare attendance) are mainly responsible of the recurrence of ARTIs, and contribute to the incidence of recurrent respiratory infections especially in the first 6 years of life [3-5].

Today, the socio-economical burden of ARTIs remains high in industrialised countries. The pharmacological cost of symptomatic drugs, antibiotics, the search for assistance by a general practitioner, hospitalisation, as well as specialist referral contribute to healthcare expenses [6-8]. Moreover, indirect costs such as parental absences from work and loss of productivity should not be neglected [9]. In consideration of current epidemiological and socio-economical data, there is a need for alternative approaches to the most well-studied and known therapies.

One functional approach to preventing and treating ARTIs is non-specifically increasing the immune response or enhancing the child’s innate defence mechanisms. Different kinds of biologically active substances – called immunostimulants – of natural and synthetic origins and with different mechanisms of action have been introduced in some countries for the prevention of ARTIs in children [10-13]. Concerning the real mechanisms of action, efficacy and safety issues have discouraged their use in different settings, in some European countries as well as in the USA. Certainly most of studies on immunostimulants were conducted many years ago and the methodological bias reported was not entirely insignificant. In our opinion, a new research input is now essential to overcome this bias, to provide new efficacy and safety data on the role of Pidotimod in preventing ARTIs in childhood.

### New evidences on mechanisms of action of Pidotimod

Recently, research focused on one of these compounds, Pidotimod, has attempted to better clarify and define its mechanism of action both *in vitro* and *in vivo*. Pidotimod (3-L-pyroglutamyl-L-thiaziolidine-4carboxylic acid) is a synthetic dipeptide molecule with immunomodulatory properties [14]. It is a highly purified molecule with high reproducibility among batches. It is rapidly absorbed by the gastrointestinal tract, with a bioavailability of 45% not influenced by food and is eliminated unmodified via renal excretory mechanisms [15]. The safety profile of Pidotimod is good; no serious adverse events were reported in human studies except for one case of suspected Henoch-Schönlein purpura [16]. However, no other association with autoimmune diseases have been reported so far.

*In vitro* studies in both animal and human specimens have shown that Pidotimod has an immunomodulatory activity on both innate and adaptive immune responses. Pidotimod induces dendritic cell (DC) maturation, upregulates the expression of HLA-DR and co-stimulatory molecules CD83 and CD86, stimulates DCs to release pro-inflammatory molecules, driving T cell proliferation and differentiation towards a Th1 phenotype, enhances natural killer cell functions, inhibits thymocyte apoptosis, and promotes phagocytosis [17-19]. More recently, Carta et al. showed that Pidotimod induced *in vitro* cellular changes that are potentially useful in enhancing the capability of the host to fight respiratory infections [20]. Through different effects on extracellular-signal-regulated kinase (ERK1/2) and nuclear factor-kappa B (NF-kB), Pidotimod increases the expression of toll-like receptor 2 proteins (surface molecules involved in the initiation of the innate response to infectious stimuli). The lack of effect on intercellular adhesion molecule (ICAM)-1 expression, the receptor for rhinovirus, and on interleukin (IL)-8 release, the potent chemotactic factor for neutrophils (usually present at sites of infection), may represent protective functions from infections. The authors concluded that Pidotimod seemed to modulate airway epithelial cell functions involved in host-virus interactions, possibly through NF-kB activation.

Studies performed using *in vivo* and *in vitro* (animal and cellular) experimental model systems are essential for identifying the biological mechanisms of action of Pidotimod, based on the assumption that these biological models have known ability to predict human responses. In spite of the encouraging results coming from *in vitro* studies, to date, *in vitro* systems do not predict all aspects of the mechanisms of action of a drug. Thus, a combination of *in vitro* and human studies is required for better characterisation of the efficacy of Pidotimod.

A recent example of bridging the gap between preclinical and clinical research was provided by Zuccotti et al. in a study conducted on children with Down syndrome, a population who frequently experiences ARTIs [21]. The authors randomised a cohort of subjects to receive Pidotimod orally or placebo and analysed immune parameters before and after the injection of seasonal 2011–2012 virosomal adjuvanted influenza vaccine. They found that the use of Pidotimod was associated with the upregulation of a number of genes involved in the activation of innate immune responses and in antimicrobial activity. Moreover, the ratio of flu-specific immunoglobulin G1/G3 (IgG1/IgG3) was skewed in Pidotimod-treated individuals, suggesting a preferential activation of complement-dependent effector mechanisms. Although preliminary, these data suggest that Pidotimod can potentiate the beneficial effect of immunisation, possibly resulting in a stronger activity of both innate and adaptive immune responses.

### Clinical issues

Up to now, clinical research on Pidotimod has mainly focused on the prevention and treatment of ARTIs in childhood. Studies conducted in the 1990s have shown that this compound seems to have a beneficial effect in children, reducing the number of ARTI, the number of days of fever, and the severity of the signs and symptoms of acute episodes [22-27]. A significant reduction in use of antibiotics, antipyretic drugs, and symptomatic drugs, and absence from school/nursery school and caregiver absenteeism was also observed [22-27]. More recently, a randomised trial has suggested that Pidotimod therapy is a reliable, simple, and safe approach to treat children with recurrent respiratory infections and it can reduce the frequency of such infections as a result of improvement of the ciliary respiratory epithelium [28].

A Cochrane meta-analysis that included all comparative randomised controlled trials that enrolled participants less than 18 years of age showed that immunostimulants reduced the incidence of ARTIs by 40% on average in susceptible children [29]. However, some bias such as the heterogeneity of subjects recruited in trials in terms of sample size, age, confounding factors (eg, concomitant asthma or allergy, number of siblings, smokers at home, seasons during the study, time and timing of attendance at daycare centre), duration of the intervention, and misused statistical tests limits the strength of conclusions of the majority of studies.

## Conclusions

Certainly the improvement of research methodology in the last 20 years and the acquired knowledge in various fields of clinical immunology should be the starting point for research on Pidotimod. Preclinical research will continue to be essential to better understand the mechanisms of action of this compound. However, *in vivo* studies, especially randomised double-blind controlled trials, are necessary to establish the role of Pidotimod in preventing ARTIs in paediatric age.

## Competing interests

The authors declare that they have no competing interests.

## Authors’ contributions

CM wrote the manuscript. GVZ critically revised the entire manuscript. Both authors read and approved the final manuscript.
